# Knowledge, Attitudes, and Behaviors about Breast Self-Examination and Mammography among Female Primary Healthcare Workers in Diyarbakır, Turkey

**DOI:** 10.1155/2016/6490156

**Published:** 2016-03-30

**Authors:** Özgür Erdem, İzzettin Toktaş

**Affiliations:** ^1^Kayapınar Family Health Centre Number 9, Diyarbakır, Turkey; ^2^Kayapınar Society Health Center, Diyarbakır, Turkey

## Abstract

*Aim*. This study aims to determine the knowledge level of the female primary healthcare workers about breast cancer and to reveal their attitude and behaviors about breast self-examination and mammography.* Methods*. This cross-sectional study was conducted on female primary healthcare workers who work in family health centres. 91% (*n* = 369) of female primary healthcare workers agreed to participate in the study. The questionnaire consisted of three parts: sociodemographic characteristics, knowledge about breast self-examination, and actual practice of breast self-examination.* Results*. The mean (SD) age of the female primary healthcare workers was 33.1 ± 6.8 (range, 20–54 years). The healthcare workers who practiced breast self-examination had significantly higher knowledge level (*P* = 0.001) than those who had not. The respondents had high knowledge level of breast self-examination; however, the knowledge level of breast cancer and mammography screen was low.* Conclusions*. While the female primary healthcare workers in this study had adequate knowledge of breast self-examination, this is not reflected in their attitudes and practices. Emphasis should be laid on breast self-examination in undergraduate and postgraduate courses for primary healthcare workers, since they are mostly involved in patient education.

## 1. Introduction

Today, cancer is one of the most serious diseases threatening human life, and therefore global burnout is gradually growing [1]. Breast cancer (BC) is the top cancer in women, both in the developed and in the developing world. The incidence of BC is increasing in the developing world due to increased life expectancy, increased urbanization, and the adoption of western lifestyles. It is estimated that, worldwide, over 508,000 women died due to BC in 2011 [2, 3]. Several risk factors for BC have been well documented; however, for the majority of women with BC, it is not possible to identify specific risk factors [4, 5]. Nevertheless, some risk reduction might be achieved with prevention. WHO promotes BC control within the context of comprehensive national cancer control programmes that are integrated into noncommunicable diseases and other related problems. Comprehensive cancer control involves prevention, early detection, diagnosis and treatment, rehabilitation, and palliative care [3].

Early detection plays a crucial role for BC. Breast self-examination (BSE), mammography, and clinical breast examination are screening methods, which are used to detect early BC. Special attention should be paid to BSE to enhance the possibility of early detection of change in breast tissue. Although BSE alone is not sufficient for early detection of BC, it allows women to be responsible for their own health, to recognize breast tissue, and to adopt preventive health behavior [6, 7]. Despite the lack of compelling evidence of its efficacy in reducing deaths from BC, systematic BSE has been recommended for the past 70 years [8]. There is no evidence on the effect of screening through BSE. However, BSE increases breast health awareness of women. Meanwhile, mammography screening is the only screening method that has proven to be effective [3].

Although the efficiency of BSE is still unclear, BSE is an important screening practice and a simple, economical, and noninvasive screening method for early detection of BC [9]. It appears that the best way to save women's lives is to increase their awareness of the potential harms of BC, to raise their level of awareness about early warning signs, risk factors, and early detection procedures for this disease [10]. Healthcare workers (HW), especially primary healthcare workers (PHW), are role models for other people in society. It is widely accepted that HW play important roles in establishing healthy behaviors. They are in a position to educate people about BC risk factors and types of screening practices and to influence behaviors that will reduce the risk of future BC morbidity and mortality. HW may also play an important role in health education by assisting people to develop a healthy behavior including BSE. Therefore, BC screening practices among HW need to be addressed.

This study aims to determine the knowledge level of the female PHW about BC, a commonly seen and treatable cancer, and to reveal their attitude and behaviors about BSE and mammography.

## 2. Materials and Methods

This descriptive, cross-sectional study was conducted among female PHW who work in family health centres (doctors, nurses, midwife, etc.) in Diyarbakır, Turkey. The population of Diyarbakır was 1,635,048 in 2014. There were 428 family health clinics in the city and 407 female HW had been working at the time. We intended to reach the entire sample; thus we contacted these female HW via e-mails and telephones after receiving ethical approval from local health authority of Diyarbakır. Potential participants were informed about the aims of the study and they were asked if they would like to volunteer for participation. They were also informed that they could withdraw from the study at any time and that all information would be kept strictly confidential. The online questionnaire form was sent to all of the female PHW, and an immediate response was requested. 91% (*n* = 369) of female PHW agreed to participate and the survey was carried out between 15 and 30 January 2015.

The questionnaire consisted of three parts: sociodemographic characteristics, knowledge about BSE, and actual practice of BSE. The questionnaire form was derived from our own experience and other published studies dealing with the same topic. The study conducted by Güleser et al. [11] was particularly taken into consideration in the preparation of the items. While the first part of the questionnaire contained sociodemographic characteristics of respondents (including age, current marital status, level of education, family history of BC, and history of breast health problems), the second part consisted of items focusing on the respondents' attitudes towards BSE and their practices of BSE as well as frequency of these practices. Finally, the third part of the questionnaire contained multiple-choice questions with one correct answer to determine the participants' knowledge level of BSE, BC, and mammography. One point was given for each correct response and zero for the wrong ones.

SPSS for Windows Version 21.0 Statistical package was used in data analysis. Data were expressed as numbers, percents, and means (SD). The scores were examined by means of the test of Kolmogorov Smirnov, and the test of homogeneity was done. As a result of this examination, no normal distribution was observed. Therefore, Mann-Whitney *U* test was done to compare the two groups, and Kruskal-Wallis analysis of variance was used to compare the multiple groups. In the comparison of the subgroups of those multiple groups which are statistically significant, Mann-Whitney *U* was conducted and the Bonferroni revision was done. The Spearman rank correlation was employed between two scores. According to statistical analyses *P* < 0.05 is considered significant.

## 3. Results

The mean (SD) age of the female PHW was 33.1 ± 6.8 (range, 20–54 years). The variables including age, level of education, personal history of breast problems, and family history of BC are not found to be significant in BSE practice (*P* > 0.05). The other sociodemographic parameters are showed at Table 1. 83.7% of the sample had at least 5-year professional experience and the mean (SD) professional experience duration of the female PHW was 10.9 ± 7.2 years (range, 6 months–37 years). There is a significant difference between the doctors' and the other (nurse, midwife, and others) HW's knowledge level (*P* = 0.026); however, there is no significant difference between the doctors' and the others' knowledge level of BSE practice (*P* > 0.05) (Table 1). There is a low correlation between age and knowledge level of HW, *r* = 0.144 (*P* = 0.005). The attitudes and practices of female PHW in BSE according to their levels of knowledge are showed in Table 2. The HW who practiced BSE had significantly higher level of knowledge (*P* = 0.001) than those who had not. In addition, the participants who practice BSE at the right time (after menstruation) had significantly higher level of knowledge (*P* = 0.001) than those who practice it at the wrong time (Table 2). The percentage of the ones older than 40 years of age who had mammography screen was 34.9% (*n* = 22) and cyst or lump was detected in 31.8% (*n* = 7) of them.  Table 3 shows the respondents' knowledge level of mammography, BC, and BSE practice. The respondents had high knowledge level of BSE; however, the knowledge level of BC and mammography screen was low.

## 4. Discussion 

BSE is an option for women starting in their 20s. Women should be informed about the benefits and limitations of BSE. Doing BSE regularly is one way for women to know how their breasts normally look and feel and to notice any changes. The goal, with or without BSE, is to report any breast-related changes to a doctor or nurse right away. At the same time, women should remember that these breast changes may not be an indicative of cancer most of the time [12].

The results of this study showed that 92.6% of the HW performed BSE, but the percentage of those who performed regular BSE was rather low (30.1%, *n* = 101). Similarly, Akpinar et al. [13] reported that 81.3% of Turkish HW (59 physicians and 302 nurses and midwives) performed BSE, but the percentage of women regularly performing this was 27.3%. In many studies conducted in Turkey [11, 14, 15], it was found that HW do perform BSE, but the rate of those doing so on a regular basis was low. These rates were even lower in women who are not HW, in the context of Turkey [9, 16]. One study, conducting a research on a large sample of female university students across 24 countries with low levels of BSE, found that 9.1% of these students practiced BSE monthly [17]. In studies performed in other countries, rates for performing BSE among HW regularly ranged from 14.4% to 46.9% [18–20]. In the present study, this finding suggests that HW are not significantly aware of the benefits of regular BSE. Thus, the regular practice of BSE among female PHW should be encouraged.

In this study, variables including age, level of education, personal history of breast problems, and family history of BC are not significant in BSE practice, which is consistent with studies of Doganer et al. [16] and Dündar et al. [21]. In contrast, it was found that age, level of education, personal history of breast problems, and family history of BC are highly significant in BSE practice in a number of studies [11, 22, 23]. The high knowledge level of BSE was determined in this study, although the knowledge levels of BC and mammography were low. The correct technique (92.1%), the frequency of women doing BSE (87.5%), and the correct time interval in relation to the menstrual cycle (90.0%) for BSE in our study were higher than those of other studies [11, 14, 19]. Güleser et al. [11] showed that, of the 52.4% who reported practicing BSE, only 48.4% of them were deemed to have an effective BSE technique, 40.2% was the frequency of women doing BSE, and 28.5% of them performed BSE with appropriate timing. In contrast to the results of the present study, Demirkiran et al. [14] found that none of the participants correctly answered the item about BSE technique and more than half the study group performed BSE with appropriate timing. Also Akpinar et al. [13] found that 55.6% of participants knew that BSE should be done in days 5–7 of the menstrual cycle.

In the present study, the HW who practiced BSE had significantly knowledge level (*P* = 0.001) than those who did not (Table 2). This result also supports some other studies [24, 25] by showing a significant relationship between individual knowledge level related to BSE and performing BSE. In contrast to our findings, it is remarkable that there was no relationship between knowing and performing BSE and having mammograms [26]. The low knowledge level about BC determined in this study is in accordance with the results of other studies [13, 20, 24]. Similarly, in our study, the low knowledge level of mammography and low screening mammography (34.9%) was detected among PHW. Consistent with the present study, screening mammography was found to be low in the previous research [27–29]. It was lower than expected especially considering the fact that participants were HW [13, 30]. There have been inadequate studies about knowledge, attitudes, and screening mammography among HW in literature.

## 5. Conclusion 

The results of this study indicate that although PHW performed BSE and had adequate knowledge, they did not perform BSE regularly and neither the knowledge level of BC nor their knowledge and attitudes about screening mammography were adequate. While the female PHW in this study had adequate knowledge of BSE, this is not reflected in their attitudes and practices. The HW, especially PHW, are role models for other people in society through their health protective behavior. The knowledge level and attitude of HW are important factors in the control of BC. It is obvious that health will improve in a society in which HW play an active role in health education.

Emphasis should be laid on BSE in undergraduate and postgraduate courses for PHW, as they are mostly involved in patient education. Also, provision of BSE educational programs is necessary for all HW to increase their knowledge, attitude, behavior, performance, and teaching of BSE. Increasing the related knowledge and application success among HW will certainly affect the women in their service area. Therefore, continuous education and in-service education for HW should be planned to improve their knowledge and experience for BSE. The results in this study also reveal that additional studies are needed to investigate the relation between BC and the mammography application among the Turkish female HW. Further research is recommended to use a larger sample size of female PHW and may include attitudes, behaviors, knowledge about BC screening methods, early detection, and implementing preventive care.

## Figures and Tables

**Table 1:** Sociodemographic characteristics of female primary healthcare workers according to knowledge level.

	*n*	%	Median (min–max)	*P* ^*∗*^
Profession				
Midwife	181	**49.1**	9 (1–15)	0.026
Nurse	112	**30.4**	9.5 (2–15)	
Doctor	57	**15.4**	10 (6–15)	
Other (emergency medical technician, dietician)	19	**5.1**	8 (2–13)	
Educational status				
High school	106	**28.7**	9 (1–15)	0.018
University	263	**71.3**	9 (1–15)	
Marital status				
Married	261	**70.7**	9 (1–15)	0.861
Single	97	**26.3**	10 (3–15)	
Divorced	8	**2.1**	10 (2–15)	
Widow	3	**0.8**	10 (7–12)	
Age				
20–39 Y	303	**82.8**	9 (1–15)	0.234
>40 Y	63	**17.2**	9 (4–15)	
Professional experience				
<10 years	189	**51.5**	9 (2–15)	0.095
>10 years	178	**48.5**	9.5 (1–15)	
Personal history of breast problems				
Yes	32	**8.7**	9 (5–14)	0.796
No	334	**91.3**	9 (1–15)	
First-degree relatives history of breast cancer				
Yes	7	**1.9**	11 (6–13)	0.796
No	358	**98.1**	9 (1–15)	
Second-degree relatives history of breast cancer				
Yes	47	**12.9**	9 (5–15)	0.699
No	318	**87.1**	9 (1–15)	
Is your menstrual period regular?				
Yes	290	**86.8**	9 (2–15)	0.227
No	44	**13.2**	9 (2–14)	

^*∗*^Significance for comparison performed by the Mann-Whitney *U* test (to compare 2 groups) or the Kruskal-Wallis test (to compare >2 groups).

**Table 2:** Attitude and practice of female primary healthcare workers in BSE according to knowledge level.

	*n*	%	Median (min–max)	*P* ^*∗*^
Did you ever perform BSE?				
Yes	339	**92.6**	9 (2–15)	0.001
No	27	**7.4**	6 (1–14)	
How often do you perform BSE?				
Once in my life	16	**4.8**	10 (3–14)	0.197
Sometimes/when it comes to mind	218	**65.1**	9 (2–15)	
Regularly, every month	101	**30.1**	10 (5–15)	
When did you start BSE?				
Age <20	32	**10.1**	9 (5–13)	0.363
Age 20	43	**13.6**	9 (4–14)	
Age >20	242	**76.3**	9 (2–15)	
When do you perform BSE?				
Before menstruation	16	**4.8**	8 (4–12)	0.001
During menstruation	13	**3.9**	9 (6–13)	
After menstruation	268	**81.0**	10 (4–15)	
When there is a complaint	26	**7.9**	8 (2–12)	
Other	8	**2.4**	9 (3–11)	
Why did not you perform BSE?				
Absence of any symptoms	16	**66.6**	6.5 (1–12)	0.877
Anxiety	3	**12.5**	6 (6–12)	
Negative family history of breast cancer	4	**16.7**	6 (3–14)	
Other	1	**4.2**	9	
Have you ever had breast exam by a doctor?				
Yes	99	**27.2**	9 (4–15)	0.309
No	265	**72.8**	9 (1–15)	
Have you ever had screening mammography? (age >40)				
Yes	22	**34.9**	10 (4–14)	0.833
No	41	**65.1**	9 (5–15)	
Do you take oral contraceptive drug or hormone replacement therapy because of menopause?				
Yes	87	**24.9**	9 (4–15)	0.864
No	262	**75.1**	9 (1–15)	

^*∗*^Significance for comparison performed by the Mann-Whitney *U* test (to compare 2 groups) or the Kruskal-Wallis test (to compare >2 groups).

**Table 3:** The female primary healthcare workers' correct answers about knowledge of BSE, breast cancer, and mammography.

The questions about BSE	The correct answers	The ratio of correct answers			
		Doctors		Other healthcare workers	
		*n* = 57	%	*n* = 312	%
At what age should women start BSE?	At the age of 20.	30	**52.6**	154	**49.4**
How often a woman should do BSE?	Once a month.	49	**86.0**	274	**87.8**
Correlating with menstruation, when should BSE be done?	After menstruation, between 7th and 10th days.	48	**84.2**	284	**91.0**
After menopause, when should BSE be done?	On the same day every month.	34	**59.6**	220	**70.5**
What is the most appropriate manual technique when doing BSE?	With the inner face of the three middle fingers.	49	**86.0**	263	**84.3**
Proper technique for BSE knowing the right technique for BSE.	Every month, both of the breast should be regularly palpated and inspected in front of a mirror.	52	**91.2**	288	**93.2**
How many minutes should be spent on each breast when practicing BSE?	For each breast 5 minutes.	22	**38.6**	115	**36.9**
What is the prevalence of breast cancer, currently?	One out of 8 women.	16	**28.1**	88	**28.2**
In which age group does the breast cancer occur more often?	After the age of 40.	39	**68.4**	149	**47.8**
In which quadrant does the breast cancer occur most often?	The upper outer quadrant.	44	**77.2**	122	**39.1**
Which type of nipple discharge should be thought to be breast cancer?	Continuous and unilateral bloody nipple secretion.	42	**73.7**	129	**41.3**
At what age should women start mammography screening?	After the age of 40.	47	**82.5**	205	**65.7**
How often should women do mammography screening?	Once every two years.	18	**31.6**	122	**39.1**

## Additional Points

The study was conducted on female PHW in Diyarbakır, Turkey. The study findings cannot be generalized to all female HW in Turkey.
